# Supplementary figures and images for: Microdialysis and CO2 sensors detect pancreatic ischemia in a porcine model
Source: PLoS One. 2022 Feb 10;17(2):e0262848. doi: 10.1371/journal.pone.0262848 (PMC8830677; doi:10.1371/journal.pone.0262848)

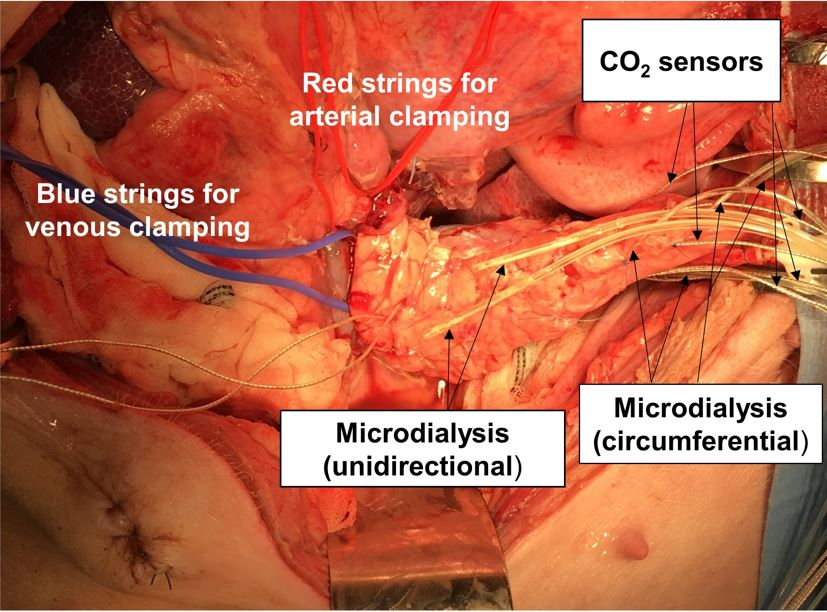

Supplement: S1 Fig — In addition the strings used for arterial and venous clamping are marked. (TIF) [file pone.0262848.s001.tif]

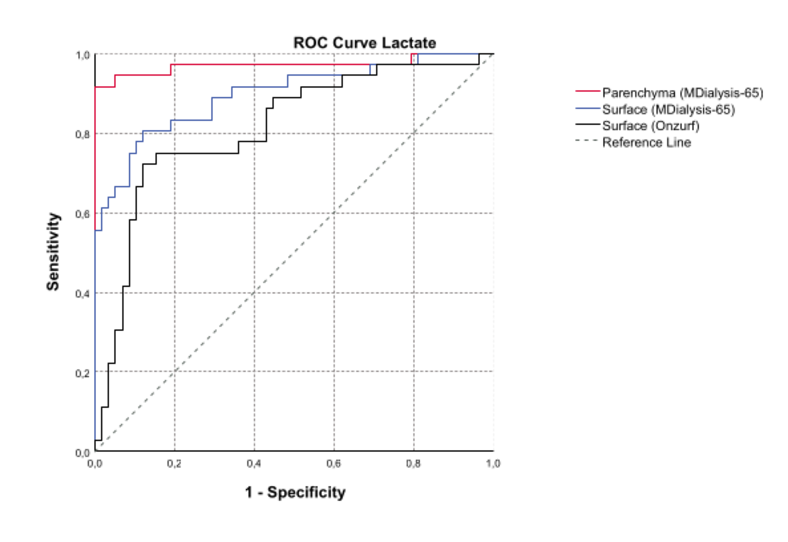

Supplement: S2 Fig — Lactate curves shown for parenchymal and surface values measured with two different microdialysis catheters, the circumferentially sampling MDialysis-65 catheter and the unidirectional sampling OnZurf catheter. (TIF) [file pone.0262848.s002.tif]

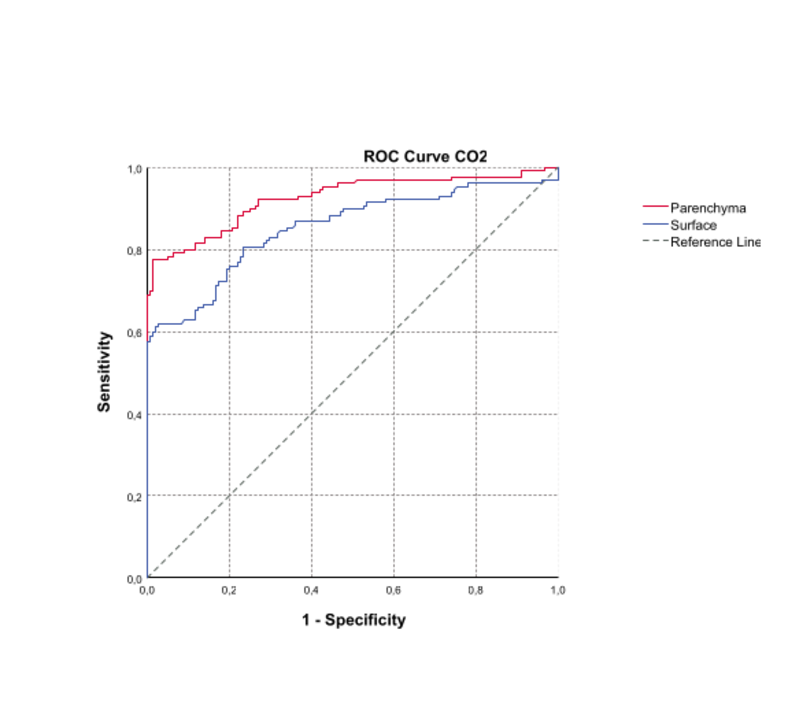

Supplement: S3 Fig — PCO2 curves from parenchymal and surface values measured with the tissue pCO2 sensors. (TIF) [file pone.0262848.s003.tif]
