# Supplementary material for: Microdialysis and CO2 sensors detect pancreatic ischemia in a porcine model
Source: PLoS One. 2022 Feb 10;17(2):e0262848. doi: 10.1371/journal.pone.0262848 (PMC8830677; doi:10.1371/journal.pone.0262848)
Supplement: S1 Table — (DOCX) [file pone.0262848.s004.docx]

## **S1 Table. Detailed information about pigs (*Sus scrofa domesticus*) used in accord with ARRIVE guidelines [1]**

| **Item** | **Explanation** |
| --- | --- |
| Species and strain | *Sus scrofa domesticus*, NOROC |
| Weight (range) | 45.5-51.5 kg |
| Age | 5 to 6 months old |
| Sex | 4 males : 4 females |
| Origin | Open-air farm (Fredrikstad, Norway) |
| Health status | No signs of infections (no diarrhoea, no infected scratching marks) |
| Housing | Pig boxes (2.4 sq.m) with raised floors in a 40 sq.m room |
| Bedding | Aspen bedding (B&K Universal Ltd, Hull, UK) |
| Housing atmosphere | Tightly regulated room temperature (20***°****C*) and humidity (55.7%), as well as 12 hours light/dark cycles. |
| Feeding | Unlimited access to tap water. Commercial pig feed was available until the night before the experiment. |
| Acclimatization period | Minimum 24 hours before the experiment. |
| Transport to OR | Sedated by intramuscular injection of ketamine (20 mg/kg), azaperone (3mg/kg), and atropine (0.02 mg/kg) in the cage. An intravenous catheter was established in an ear vein and sedation deepened using pentobarbital (1– 2 mg/kg) and morphine (0.5 mg/kg). |
| OR atmosphere | Sterile and stable environment with regulated temperature (22*°C*) and humidity (50-60 %). |
| Temperature | A urinary catheter with a temperature probe was inserted via a cystotomy into the urinary bladder. If the animals´ bladder temperature increased > 39.0 *°C* they were actively cooled using cold intravenous fluid and external ice packages. |
| Anaesthesia | Balanced anaesthesia with Isoflurane 1.0-2.0 % end tidal concentration and morphine infusion of 1-2 mg/kg/h. Anaesthesia depth was regularly evaluated by pinching of interdigital skin, reactions of movement, pulse or blood pressure increase monitored. Muscle relaxants were not administered. |
| Respiration | Standard respirator (Aespire View, Datex-Ohmeda/GE Healthcare, Finland). Tidal volumes were set to 7-8 mL/kg, respiratory rate to 18-22 breaths/min, an inspiratory/expiratory time ratio of 1:2, a positive end-expiratory pressure (PEEP) of 5 cm H_2_O and a fraction of inspired oxygen (FiO_2_) of 0.3-0.5 aiming at a PaCO_2_ of approximately 6 kPa to keep pH normal and a PaO_2_ >8 kPa. |
| Hemodynamic monitoring | Central venous access (7 Fr, 20 cm) via the right internal jugular vein. An arterial line (16 G Secalon-T) was inserted in the right carotid artery. A crystalloid infusion (Ringer acetate or NaCl 0.9% of 20-30 ml/kg/h) was administered to keep the hemoglobin concentration stable and to maintain a mean arterial blood pressure above 50 mmHg. Monitoring with 3-lead electrocardiography, pulse oximetry, arterial blood pressure and central venous pressure was applied. |
| Glucose control | Glucose 5-10% was infused at a rate of 20-40 mL/h to keep the blood glucose concentration between 5-8 mmol/L. |
| Euthanasia | Bolus of Pentobarbital (1000 mg), Morphine (50-100 mg) and Potassium chloride (70-100 mmol). |

OR: operation room, iv: intravenous

1. Kilkenny C, Browne WJ, Cuthill IC, Emerson M, Altman DG. Improving bioscience research reporting: the ARRIVE guidelines for reporting animal research. PLoS Biol. 2010;8(6):e1000412. Epub 2010/07/09. doi: 10.1371/journal.pbio.1000412. PubMed PMID: 20613859; PubMed Central PMCID: PMCPMC2893951.
