# Supplementary material for: Microdialysis and CO2 sensors detect pancreatic ischemia in a porcine model
Source: PLoS One. 2022 Feb 10;17(2):e0262848. doi: 10.1371/journal.pone.0262848 (PMC8830677; doi:10.1371/journal.pone.0262848)
Supplement: S2 Table — (DOCX) [file pone.0262848.s005.docx]

**Table S2. Number of microdialysis catheters with results from each location /Number of catheters placed**

|  |  | **Ischemic part of pancreas (tail of pancreas)** | | | | | **Attempted non-ischemic part of pancreas (caput of pancreas)** | | | | |
| --- | --- | --- | --- | --- | --- | --- | --- | --- | --- | --- | --- |
|  | **Catheter type** | **MD** | **MD** | **MD(O)** | **CO_2_** | **CO_2_** | **MD** | **MD** | **MD(O)** | **CO_2_** | **CO_2_** |
| **Pig nr** | **Localisation of catheter** | **P** | **S** | **S** | **P** | **S** | **P** | **S** | **S** | **P** | **S** |
| 1 |  | 2/2 | 2/2 | 0 | 2/2 | 1/1 | 1 | 1 | 1 | 1 | 0 |
| 2 |  | 2/2 | 2/2 | 1/1 | 2/2 | 1/1 | 1 | 1 | 1 | 1 | 1 |
| 3 |  | 1/2 | 2/2 | 1/1 | 2/2 | 1/2 | 1 | 1 | 1 | 1 | 1 |
| 4 |  | 2/2 | 1/2 | 1/1 | 1/2 | 1/2 | 1 | 1 | 1 | 1 | 1 |
| 5 |  | 2/2 | 1*/2 | 2/2 | 2/3 | 2/2 | - | - | - | - | - |
| 6 |  | 2/2 | 2/2 | 2/2 | 3/3 | 2/2 | - | - | - | - | - |
| 7 |  | 2/2 | 2/2 | 2/2 | 2/3 | 1/2 | - | - | - | - | - |
| 8 |  | 2/2 | 2/2 | 2/2 | 3/3 | 1/2 | - | - | - | - | - |

MD, microdialysis (circumferential); MD (O), microdialysis Onzurf (unidirectional); P, parenchymal; S, surface. The supposed non-ischemic part of the pancreas, got ischemic and measurements here were abandoned after the first four pigs, and none of the results from this location is presented in the article. * This catheter stopped working after arterial occlusion and we have no data from the venous occlusion from this catheter.
