# Supplementary material for: Microdialysis and CO2 sensors detect pancreatic ischemia in a porcine model
Source: PLoS One. 2022 Feb 10;17(2):e0262848. doi: 10.1371/journal.pone.0262848 (PMC8830677; doi:10.1371/journal.pone.0262848)
Supplement: S3 Table — (DOCX) [file pone.0262848.s006.docx]

**S3 Table. Correlation coefficients (R) for lactate between microdialysis catheters placed in the same location (parenchyma or surface)**

|  | **Parenchyma catheter**  **1 vs 2** | | **Surface (circum-**  **ferential)**  **catheter 1 vs 2** | | **Surface (uni-**  **directional)**  **catheter 1 vs 2** | |
| --- | --- | --- | --- | --- | --- | --- |
| Pignr | R | *p*-value | R | *p*-value | R | *p*-value |
| 1 | 0.79 | <0.001 | 0.79 | <0.001 | n.a |  |
| 2 | 0.86 | <0.001 | 0.92 | <0.001 | n.a |  |
| 3 | n.a |  | 0.56 | 0.0036 | n.a |  |
| 4 | 0.87 | <0.001 | n.a |  | n.a |  |
| 5 | 0.76 | <0.001 | n.a |  | 0.54 | 0.0035 |
| 6 | 0.88 | <0.001 | 0.65 | 0.0002 | 0.32 | 0.115 |
| 7 | 0.96 | <0.001 | 0.89 | <0.001 | 0.87 | <0.001 |
| 8 | 0.89 | <0.001 | 0.92 | <0.001 | 0.97 | <0.001 |

n.a., results from two catheters of the same location not available.
