# Supplementary material for: Microdialysis and CO2 sensors detect pancreatic ischemia in a porcine model
Source: PLoS One. 2022 Feb 10;17(2):e0262848. doi: 10.1371/journal.pone.0262848 (PMC8830677; doi:10.1371/journal.pone.0262848)
Supplement: S4 Table — (DOCX) [file pone.0262848.s007.docx]

**S4 Table. Correlation coefficients (R) for pCO_2_ between tissue pCO_2_ sensors placed in the same location (parenchyma or surface)**

|  | **Parenchyma**  **sensor 1 vs 2** | | **Surface**  **sensor 1 vs 2** | |
| --- | --- | --- | --- | --- |
| Pignr | R | *p*-value | R | *p*-value |
| 1 | 0.81 | <0.001 | n.a |  |
| 2 | 0.63 | <0.001 | n.a |  |
| 3 | 0.85 | <0.001 | n.a |  |
| 4 | 0.90 | <0.001 | n.a |  |
| 5 | 1.0 | <0.001 | 0.77 | <0.001 |
| 6 | 0.93 | <0.001 | 0.73 | <0.001 |
| 7 | 0.88 | <0.001 | n.a |  |
| 8 | 0.91 | <0.001 | n.a. | n.a. |

n.a., results from two catheters of the same location not available.
